# Relationship between ABO blood group and lymph node metastasis in colon cancer: a retrospective cohort study

**DOI:** 10.1186/s12957-025-04179-1

**Published:** 2026-01-27

**Authors:** Camilo Ramírez-Giraldo, Hakim Jaber, Nicolò Fabbri, Alberto Cataldi, Gianluca Lodi, Bruno Cirillo, Carlo Feo, Antonio Pesce

**Affiliations:** 1 Department of Surgery, Universidad del Rosario – Hospital Universitario Mayor Méderi, Bogotá, Colombia; 2 Faculty of Medicine and Surgery, University of Ferrara, Ferrara, Italy; 3 Department of Surgery, Azienda Unità Sanitaria Locale Ferrara, University of Ferrara, Via Valle Oppio, 2, Lagosanto, 44023 FE Italy; 4 Blood Transfusion Service, Arcispedale S. Anna, Via Aldo Moro, Cona, Ferrara, 8 - 44124 Italy; 5 Department of Surgery, Sapienza University of Rome, Viale Regina Elena 324, Rome, 00161 Italy

**Keywords:** Colon neoplasm, Lymphatic metastasis, ABO blood group system, Microsatellite instability, Prognostic factors

## Abstract

**Purpose:**

Colorectal cancer is the third most common cancer and the second leading cause of cancer-related deaths worldwide, according to GLOBOCAN 2022. Lymph node metastasis is a well-recognized prognostic factor in colorectal cancer. While the relationship between ABO blood group, Rhesus (Rh) type, and lymphatic spread has been studied in other gastrointestinal tumors, limited research exists on colorectal cancer. This study primarily aimed to investigate the association between lymph node metastasis and ABO blood group, as well as the relationship between microsatellite instability (MSI) and ABO blood group.

**Methods:**

. A retrospective observational cohort study was conducted, including all patients who underwent elective colorectal resections with curative intent for malignant colorectal tumors between March 2017 and March 2023. Eligible patients had documented ABO blood group and Rh type, along with pathology reports from surgical specimens.

**Results:**

. The study included 270 patients, with a median age of 74.5 years-old. The cohort was predominantly female (50.4%). Lymph node metastasis was observed in 156 patients (57.7%). A binary logistic regression model identified factors associated with lymphatic spread: rectal tumor location (OR: 3.85, 95% CI: 1.14–15.60), poorly differentiated tumors (OR: 6.84, 95% CI: 1.37–53.80), invasion depth T3 (OR: 4.88, 95% CI: 1.72–16.90) and T4 (OR: 16.20, 95% CI: 4.78–65.60), and extramural vein invasion (OR: 7.17, 95% CI: 1.37–53.80). Notably, the AB blood group (OR: 0.12, 95% CI: 0.01–0.65) was associated with a lower likelihood of lymph node metastasis, suggesting a potential protective effect. A separate binary logistic regression analysis evaluating factors related to MSI found no statistically significant associations, including for ABO blood group and Rh antigen.

**Conclusions:**

Our findings suggest that the AB blood group is associated with a reduced likelihood of lymph node metastasis compared to other blood groups. However, the existing literature on the relationship between blood group and lymph node metastasis is inconsistent. Further research is necessary to clarify the prognostic role of ABO blood group in colorectal cancer.

## Introduction

Colorectal cancer is the third most common malignancy worldwide and the second leading cause of cancer-related death, according to GLOBOCAN 2022 [1]. Its development is influenced by numerous modifiable and non-modifiable risk factors, including advanced age, male sex, inflammatory bowel disease, and a family history of colorectal cancer [2]. Surgery remains the cornerstone of treatment, and current guidelines recommend retrieving at least 12 lymph nodes to ensure accurate lymphatic staging [3–5]. However, extending the extent of lymphadenectomy solely to increase lymph node yield is associated with increased morbidity and has not demonstrated oncological benefit in contemporary colorectal surgery [6].

Lymph node metastasis is a well-established prognostic factor in colorectal cancer [3–7]. Its presence is thought to reflect alterations in tumor–host interactions, including changes in adhesion molecules such as soluble intercellular adhesion molecule-1 (sICAM-1), E-selectin, and P-selectin, which have been reported to be lower in individuals with non-O blood groups [8]. Moreover, several studies have identified ABO blood group antigens on the surface of enterocytes, suggesting that variations in their expression may influence tumor biology and potentially affect the pattern of lymphatic spread [7, 9]. In addition, some evidence indicates that ABO blood group may be associated with the frequency of microsatellite instability (MSI), particularly with O blood group and Rh antigen positivity [9]; however, the available studies remain limited and inconclusive.

MSI is a hallmark of mismatch repair deficiency in colorectal cancer and represents another key biological characteristic with important prognostic and therapeutic implications [10]– [11].

While the relationship between ABO blood group, Rhesus (Rh) type, lymph node metastasis, and survival has been investigated in other gastrointestinal malignancies, data specific to colorectal cancer are scarce. This gap highlights the need to clarify the potential prognostic role of blood group antigens in this disease.

## Materials and methods

### Study design

A retrospective observational cohort study was conducted, including all Caucasian patients who underwent elective laparoscopic colorectal resections with curative intent for malignant colorectal tumors between March 2017 and March 2023.

Inclusion criteria were: documented ABO blood group and Rh factor, along with available pathology reports from the surgical specimen.

Exclusion criteria included: colonic resections performed for non-neoplastic diseases; rare subtypes of malignant colorectal tumors (e.g., lymphoma, melanoma, and neuroendocrine carcinoma), due to their distinct biological behavior; patients with mid or low rectal cancer, including those who received neoadjuvant therapy; emergency surgeries; and cases with missing ABO blood group data.

All data were collected in an anonymized database.

The study protocol was approved by the local Ethics Committee, Comitato Etico Area Vasta Emilia Centro – CE-AVEC, under ID: 194/2025/Oss/AUSLFe and was conducted in accordance with the Declaration of Helsinki. This study is reported following the Strengthening the Reporting of Observational Studies in Epidemiology (STROBE) guidelines [12].

The following variables were analyzed: age, sex, type of surgery, tumor location and size, histological grade, depth of invasion, extramural venous invasion, lymph node metastasis, final histopathological report, ABO blood group, Rh factor, microsatellite instability (MSI) status including MSI-H subtypes, and K-RAS and B-RAF mutations. Tumor size was determined by pathological examination.

### Statistical analysis

Demographic, clinical, and surgical variables were described. Categorical variables were presented as proportions, while continuous variables were summarized as medians with their respective interquartile range (IQR). A bivariate analysis was conducted to compare differences based on nodal metastasis (N+), using the chi-squared test for categorical variables and the Mann–Whitney U test for continuous variables. Binary logistic regression was performed to identify factors associated with N+, including primary tumor site, tumor differentiation, invasion depth (T status), extramural vein invasion, blood group, and Rh antigen. Additionally, an exploratory analysis was conducted among patients assessed for microsatellite instability (MSI), comparing them with those who had microsatellite stability (MSS). The percentages of missing data were as follows: tumor localization (0.4%), tumor size (4.8%), tumor differentiation (2.6%), and extramural venous invasion (9.6%). Missing values were assumed to be missing at random and were handled using multiple imputation by predictive mean matching. Five imputed datasets were generated. Descriptive baseline characteristics are presented using one representative imputed dataset for clarity. In contrast, all multivariable logistic regression analyses were conducted across the five imputed datasets, and parameter estimates (coefficients, odds ratios, 95% confidence intervals, and p-values) were pooled according to Rubin’s rules to appropriately account for the uncertainty associated with missing data. A p-value < 0.05 was considered statistically significant. All analyses were performed using R (version 2023.12.1 + 402).

## Results

This study included 270 patients, with a median age of 74.5 years (IQR: 66.0–82.1). The cohort was predominantly female (50.4%). The average number of lymph nodes sampled for each patient was 16.2 (median 13.0; range 1–65). Lymph node metastasis was observed in 156 patients (57.7%).

Table 1 presents the demographic, clinical, and pathological characteristics stratified by lymph node metastasis.

**Table 1:** Demographic, clinical, and pathological characteristics stratified by lymph node metastasis

	*N* Overall (%)	Lymph node negative(*n* = 156)	Lymph node positive (*n* = 114)	*p* value
Age(median)(IQR)(years)	74.57 (66.03–82.15)	75.47 (66.04–82.72)	73.78 (66.13–80.70)	0.275*
Sex				
Female	136 (50.4)	79 (50.6)	57 (50.0)	1.000
Male	134 (49.6)	77 (49.4)	57 (50.0)	
Primary tumor site				
Colon	243 (90.0)	148 (94.9)	95 (83.3)	**0.005**
Rectum	17 (6.3)	4 (2.6)	13 (11.4)	
Recto-sigmoid junction	10 (3.7)	4 (2.6)	6 (5.3)	
Tumor size (median)(IQR)(cm)	4.00 (3.00–5.50)	3.90 (2.50–6.00)	4.25 (3.00–5.50)	0.105
Tumor differentiation				
Well differentiated	12 (4.4)	10 (6.4)	2 (1.8)	**0.001**
Moderately differentiated	201 (74.4)	124 (79.5)	77 (67.5)	
Poorlydifferentiated	57 (21.2)	22 (14.1)	35 (30.7)	
Histology				
Adenocarcinoma not-otherwise-specified (NAS)	174 (64.4)	99 (63.5)	75 (65.8)	0.343
Mucinous	85 (31.5)	53 (34.0)	32 (28.1)	
Signet ring cell	7 (2.6)	2 (1.3)	5 (4.4)	
Medullary	4 (1.5)	2 (1.3)	2 (1.8)	
Synchronic				
Yes	13 (4.8)	8 (4.1)	5 (4.4)	1.000
No	257 (95.2)	148 (94.9)	109 (95.6)	
Invasion depth				
T1 T2 T3 T4	40 (14.8) 36 (13.3) 143 (53.0) 51 (18.9)	35 (22.4) 28 (17.9) 79 (50.6) 14 (9.0)	5 (4.4) 8 (7.0) 64 (56.1) 37 (32.5)	**< 0.001**
Invasion extramural veins				
No Yes	242 (89.6) 28 (10.4)	152 (97.4) 4 (2.6)	90 (78.9) 24 (21.1)	**< 0.001**
Mutational status				
No mutation K-RAS mutant B-RAF mutant Unknown	13 (4.8) 9 (3.3) 43 (15.9) 205 (75.9)	6 (3.8) 3 (1.9) 28 (17.9) 119 (76.3)	7 (6.1) 6 (5.3) 15 (13.2) 86 (75.4)	0.275
Blood group				
0-blood group A-blood group B-blood group AB-blood group	120 (44.4) 108 (40.0) 28 (10.4) 14 (5.2)	71 (45.5) 57 (36.5) 16 (10.3) 12 (7.7)	49 (43.0) 51 (44.7) 12 (10.5) 2 (1.8)	0.128
Rh antigen				
Rh+ Rh-	240 (88.9) 30 (11.1)	137 (87.8) 19 (12.2)	103 (90.4) 11 (9.6)	0.647
MSS MSI	214 (79.3) 56 (20.7)	123 (78.8) 33 (21.2)	91 (79.8) 23 (20.2)	0.965

Bold values indicate statistically significant p values (*p* < 0.05)

p values were obtained using the chi-squared test

*p values were obtained using the Mann–Whitney test

In the bivariate analysis, rectal tumor location, larger tumor size, poorer tumor differentiation, greater tumor invasion depth, and extramural venous invasion were significantly associated with positive lymph node status. In contrast, blood group and Rh antigen did not differ between the groups. We also evaluated the association between ABO blood type and tumor differentiation, and no statistically significant differences were observed (*p* = 0.715).

We performed a binary logistic regression analysis to identify factors associated with lymph node metastasis. The model revealed the following significant associations: rectal tumor location (OR: 3.79, 95% CI: 1.07–13.4), invasion depth classified as T3 (OR: 5.04, 95% CI: 1.66–15.3) or T4 (OR: 18.0, 95% CI: 4.93–65.7), and extramural venous invasion (OR: 4.75, 95% CI: 1.09–20.7). Additionally, the AB blood group was identified as a protective factor against lymphatic spread. These findings are summarized in Table 2.

**Table 2:** Binary logistic regression model to identify factors related to lymph node metastasis

	OR (CI 95%)	*p* value
Primary tumor site		
Colon Rectum Recto-sigmoid junction	Reference 3.79 (1.07–13.4) 2.46 (0.55–11.1)	**0.039** 0.240
Tumor differentiation		
Well differentiated (G1) Moderately differentiated (G2) Poorly differentiated (G3)	Reference 1.44 (0.27–7.50) 4.11 (0.73–23.2)	0.667 0.109
Invasion depth		
T1 T2 T3 T4	Reference 2.68 (0.70–10.2) 5.04 (1.66–15.3) 18.0 (4.93–65.7)	0.148 **0.005** **< 0.001**
Invasion extramural veins		
No Yes	Reference 4.75 (1.09–20.7)	**0.039**
Blood group		
AB-blood group 0-blood group A-blood group B-blood group	Reference 8.11 (1.24–53.2) 11.5 (1.70–77.1) 9.70 (1.24–75.9)	**0.029** **0.012** **0.031**
Rh antigen		
Rh- Rh+	Reference 1.49 (0.59–3.80)	0.398

Bold values indicate statistically significant p values (*p* < 0.05)

Of the 270 patients, 56 were identified as having MSI and 214 microsatellite stable (MSS). In the bivariate analysis, no statistically significant differences were observed between the MSI and MSS groups with ABO blood groups and Rh antigen, as shown in Table 3. However, it is noteworthy that MSS patients more frequently exhibited poor prognostic features, including T4 invasion depth, lymphatic involvement, and extramural venous invasion. Moreover, in the binary logistic regression analysis evaluating factors associated with MSI, no statistically significant associations were found between either blood group or Rh antigen and MSI status.

**Table 3:** Demographic, clinical, and pathological characteristics stratified by microsatellite instability

	*N* overall (%)	MSI group (*n* = 56)	MSS group (*n* = 214)	*p* value
Age (median)(IQR)(years)	74.57 (66.03–82.15)	75.65 (69.90–81.04)	74.10(65.85–82.34)	0.654*
Sex				
Female Male	136 (50.4) 134 (49.6)	31 (55.4) 25 (44.6)	105 (49.1) 109 (50.9)	0.491
Tumor site				
Colon Rectum Recto-sigmoid junction	243 (90.0) 7 (6.3) 0 (3.7)	55 (98.2) 1 (1.8) 0 (0.0)	188 (87.9) 16 (7.5) 10 (4.7)	0.066
Tumor size (median)(IQR)(cm)	4.00 (3.00–5.50)	4.50 (3.00–6.12)	4.00 (3.00–5.50)	0.193*
Tumor differentiation				
Well differentiated Moderately differentiated Poorlydifferentiated	12 (4.4) 201 (74.4) 57 (21.2)	1 (1.8) 34 (60.7) 21 (37.5)	11 (5.1) 167 (78.0) 36 (16.8)	0.908
Invasion depth				
T1 T2 T3 T4	40 (14.8) 36 (13.3) 143 (53.0) 51 (18.9)	1 (1.8) 13 (23.2) 33 (58.9) 9 (16.1)	39 (18.2) 23 (10.7) 110 (51.4) 42 (19.6)	**0.003**
Histology				
Adenocarcinoma not- otherwise-specified (NAS) Mucinous Signet ring cell Medullary	174 (64.4) 85 (31.5) 7 (2.6) 4 (1.5)	18 (32.1) 30 (53.6) 4 (7.1) 4 (7.1)	156 (72.9) 55 (25.7) 3 (1.4) 0 (0.0)	**< 0.001**
Synchronic				
Yes No	13 (4.8) 257 (95.2)	4 (7.1) 52 (92.9)	9 (4.2) 205 (95.8)	0.573
Lymph node status				
Negative Positive	156 (57.8) 114 (42.2)	33 (58.9) 23 (41.1)	123 (57.5) 91 (42.5)	0.965
Invasion extramural veins				
No Yes	242 (89.6) 28 (10.4)	52 (92.9) 4 (7.1)	192 (89.7) 22 (10.7)	0.650
Mutational status				
No mutation K-RAS mutant B-RAF mutant Unknown	13 (4.8) 9 (3.3) 43 (15.9) 205 (75.9)	11 (19.6) 2 (3.6) 31 (55.4) 12 (21.4)	2 (0.9) 7 (3.3) 12 (5.6) 193 (90.2)	**< 0.001**
Blood group				
0-blood group A-blood group B-blood group AB-blood group	120 (44.4) 108 (40.0) 28 (10.4) 14 (5.2)	28 (50.0) 21 (37.5) 5 (8.9) 2 (3.6)	92 (43.0) 87 (40.7) 23 (10.7) 12 (5.6)	0.778
Rh antigen				
Rh+ Rh-	240 (88.9) 30 (11.1)	52 (92.9) 4 (7.1)	26 (12.1) 188 (87.9)	0.411

p values were obtained using the chi-squared test

*p values were obtained using the Mann–Whitney test

## Discussion

There have been few studies on the relationship between ABO blood groups and lymph node metastasis of malignant diseases. In 1986, Halvorsen TB from Norway retrospectively analyzed 747 patients with single colonic adenocarcinomas. No differences were observed in ABO blood group distribution concerning tumor stage; however, he noted that metastases into regional lymph nodes were more common among Rh-positive patients [13]. Based on studies on melanoma [14] where Rh-negative patients exhibited significantly higher natural killer cell activity against target cells, Halvorsen TB hypothesized that Rh-negative patients with colorectal cancer might have a better immunological response and consequently a lower lymph node spread [13]. ABO blood group antigens are expressed on red blood cells, endothelial cells, and various tissues, including colonic cells. The immune system’s recognition of these antigens could play a role in the immune surveillance of colorectal cancer. A few years later, Slater G et al., after analyzing 838 patients with colorectal cancer, found no differences in stage distribution regarding ABO blood groups and Rh antigens. However, he observed that all patients with synchronous cancers were Rh-positive [15]. In our study, no differences were found in Rh positive in comparison with Rh negative patients. Our findings suggest that patients with the AB blood group have a lower likelihood of lymphatic metastasis compared to other blood groups, which might be associated with a better prognosis in colorectal cancer patients. This aligns with findings from Cao X et al., who reported improved survival rates in AB blood group patients compared to other blood groups [16]. Among 1,555 colon cancer patients who underwent curative intent surgery, the mean overall survival of patients with blood type AB was 113.9 months, compared to 106.1 months for patients with non-AB blood types [16]. The mechanisms underlying the reduced likelihood of lymph node metastasis remain unclear; however, several hypotheses can be proposed. One possible explanation is that the loss of antigens A and B may increase metastatic spread to lymph nodes.

A key element in this process involves the fucosyltransferase genes FUT2 and FUT3, which influence the expression of histological blood group antigens (HBGAs) on the gastrointestinal mucosa and in body secretions. FUT2, located on chromosome 19, encodes α−1,2-fucosyltransferase, an enzyme required for the secretion of soluble ABH and Lewis antigens in mucosal tissues and secretory glands [17].

Both ABH and Lewis antigens serve not only as binding targets for gut microorganisms but also as carbon sources for these microbes. Therefore, the gut microbiota is shaped in part by FUT2 and FUT3 activity, which regulates HBGA presentation in the gastrointestinal tract [17, 18]. In individuals carrying a functional FUT2 gene (“secretors”), ABH expression is abundant in the stomach and small intestine but decreases progressively from the proximal to the distal colon [19–22]. This gradient could help explain the stronger association between rectal tumors and lymph node metastasis.

In contrast, FUT3 encodes α-(1,3/4)-fucosyltransferase, which synthesizes the Lewis a antigen and uses the H antigen (produced via FUT2) to form Lewis b [20]. The Lewis b antigen is mostly expressed in the proximal colon and absent in the distal colon, whereas Lewis a is consistently expressed throughout the colon [20, 23]. These patterns support the concept of a plurigenic regulation of glycan expression, particularly in individuals with the AB blood group, who may exhibit more stable enterocytic antigen expression.

Importantly, some advanced tumors frequently lose ABH antigen expression, and the incomplete glycan synthesis typical of cancer cells leads to reduced expression of antigens A and B [24, 25]. Therefore, the presence of A and B antigens on colonic tumor cells may be associated with a more favorable prognosis, as their loss could facilitate metastatic progression under comparable clinical conditions.

Moreover, we observed that rectal tumor site, poorly differentiated tumors, T3 and T4 invasion depth, and extramural venous invasion were significantly associated with lymph node metastasis. These factors are well-established prognostic indicators and are strongly linked to a higher probability of lymphatic spread [26].

The relationship between blood group, survival, and lymph node metastasis has also been explored in other gastrointestinal malignancies [27–30]. For instance, Qiu MZ et al. examined lymphatic invasion and survival rates in 474 patients with confirmed gastric cancer, finding that patients with blood group O had a lower rate of angiolymphatic invasion. No significant differences in survival were observed among ABO blood groups, although patients with blood types B and O appeared to have higher survival rates compared to those with blood types A and AB [30]. The discrepancy in the lower survival rate of AB blood group patients with gastric cancer can be explained by the differing behavior and expression of ABO antigens in gastric epithelial cells, as well as the role of the microbiota in colon cancer. The interplay between the microbiota and ABO antigen expression in colonic cells influences microbial colonization, immune responses, and potentially disease outcomes [31, 32]. The presence of ABH and Lewis antigens modulates microbial niches, while dysbiosis and changes in antigen expression in diseases like colorectal cancer may have significant diagnostic and therapeutic implications. However, Rashid G et al., in a study of 246 patients with confirmed colorectal cancer, reported no significant association between ABO blood group and 3-year survival rates during follow-up [33].

Indeed, we did not find any specific blood group to be associated with a higher proportion of microsatellite instability. Karaoglan B et al. reported that MSI-H was more frequently observed in patients with the O blood group and Rh + phenotype [9]. This finding could be relevant, as MSI is known to influence prognosis, with MSI-positive patients experiencing different outcomes compared to those with microsatellite stable (MSS) tumors [9–11].

The exact functions of ABO blood group antigens remain unclear. While the absence of ABO antigens is not directly linked to disease, susceptibility to certain conditions has been associated with specific ABO phenotypes. For example, an association between the A blood group and gastric cancer has been reported [34, 35]. The literature on the relationship between blood group, survival, and lymph node metastasis is contradictory. Further studies are needed to clarify the potential prognostic role of blood group in colorectal cancer.

However, we believe that a potential relationship between blood type and lymph node metastasis could have some important implications for clinical practice, particularly in surgical oncology. Early-stage cancers in high-risk blood types might warrant a more extensive evaluation of regional lymph nodes, and surgeons may adjust the extent of lymphadenectomy based on blood type-associated risk. Overall, this association could help guide tailored treatment and surveillance strategies. Future molecular biology research could clarify the role of ABO blood groups in the different behavior of colorectal cancer.

### Limitations and strength

A key limitation of this study is its retrospective design, along with its nature as a single-center study with a relatively small sample size. Another limitation of this study is the absence of data on perineural invasion and tumor budding, both recognized prognostic factors associated with lymph node metastasis in colorectal cancer. Their omission may introduce residual confounding and should be taken into account when interpreting the associations observed. Moreover, patient survival was not analyzed in this study, as the focus was specifically limited to exploring lymph node metastasis in colorectal cancer patients. A strength of this study is that it is one of the few recent studies analyzing a possible correlation between ABO blood group and lymph node metastasis in colorectal cancer patients.

## Conclusion

Our findings suggest that the AB blood group is associated with a reduced likelihood of lymph node metastasis compared to other blood groups. ABO blood groups could potentially have a role in lymph node metastasis in colorectal cancer, but this relationship is still not well understood and requires further research to clarify any associations.

## Data Availability

The data that support the findings of this study are available on request from the corresponding author.
